# Exuberant Intratracheal Granuloma

**DOI:** 10.1155/2021/6697478

**Published:** 2021-02-22

**Authors:** Emelia Stuart, Michael Armaneous, David Bracken, Kayva Crawford, Andrew M. Vahabzadeh-Hagh

**Affiliations:** ^1^University of California San Diego Health Sciences, 9500 Gilman Dr., La Jolla, CA 92093, USA; ^2^Touro University Nevada College of Osteopathic Medicine, 874 American Pacific Dr. #8801, Henderson, NV 89014, USA; ^3^University of California San Diego Health Sciences, Department of Surgery, Division of Otolaryngology, 9300 Campus Point Drive Suite LL-A, La Jolla, CA 92037, USA

## Abstract

**Background:**

Upper airway granulomas are commonly encountered benign masses and are a result of pronounced tissue reactivity to localized respiratory mucosal trauma. The mechanism of injury to respiratory epithelium is most commonly iatrogenic and associated with intubation or indwelling tracheostomy. *Case Report*. A 40-year-old obese female with a history of multiple intubations, poorly controlled diabetes mellitus type II, and history of tracheal stenosis presented with sudden onset respiratory distress requiring intubation at an outside hospital. Direct laryngoscopy revealed a rapidly forming transglottic tissue mass, measuring 5.0 × 2.2 × 0.8 cm. The following case represents an unusual exception to our experience with granulomas given its rapidity of onset and migration of tissue around the endotracheal tube. Discussion. Laryngeal erythema and granulation formation are expected postintubation findings in most patients; however, the large size of granuloma tissue and rapid onset of symptoms in this case make it remarkable. Our patient had multiple risk factors for postintubation stenosis: female sex, poorly controlled diabetes, hypertension, obesity, and multiple prior intubations for periods lasting longer than forty-eight hours.

**Conclusion:**

Our case highlights a rare laryngeal finding of a large granulation tissue mass causing sudden onset airway obstruction.

## 1. Introduction

Vocal process granulomas are benign lesions found in the posterior glottis [1, 2]. Rather than true granulomas, these collections of granulation or fibrotic tissue heaped beneath injured squamous epithelium represent a reactive or reparative process [2]. The most common causes include laryngopharyngeal reflux (LPR), vocal abuse, and intubation trauma [3–5]. Risk factors associated with the development of laryngeal granulomas after intubation include female sex, prolonged intubation, traumatic intubation, inappropriate tube size, high cuff pressures, and presence of a concurrent nasogastric tube. However, no single risk factor for progression to chronic granuloma has been consistently identified [3, 6, 7]. Some techniques put forward to reduce the occurrence including use of high volume and low-pressure cuffs and choosing appropriate tube size to minimize contact injury. These lesions most commonly occur on the vocal process of the arytenoid cartilage, where the endotracheal tube exerts pressure. The vocal process is also vulnerable due to its thin mucoperichondrial covering and frequent, powerful impacts during vocal cord adduction [4]. In addition to granuloma formation, prolonged intubation is also the leading cause of tracheal stenosis. The most common site for postintubation tracheal stenosis occurs at the cuff site and is attributed to localized tissue ischemia secondary to pressure [8]. Postintubation tracheal stenosis recurs in some cases, but the rate of recurrence and need for intervention varies widely between patients.

## 2. Case Description

**Figure 1:**
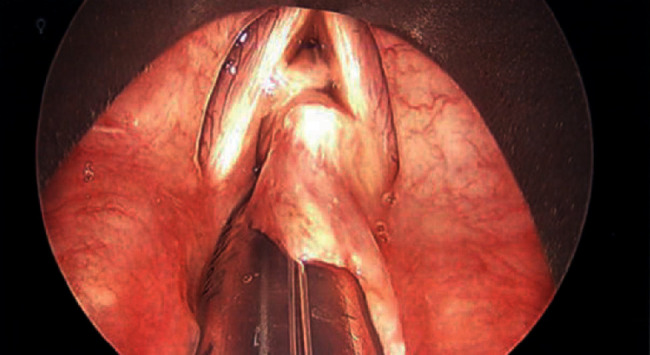
Sessile soft tissue mass arising from the subglottic space adherent to endotracheal tube.

**Figure 2:**
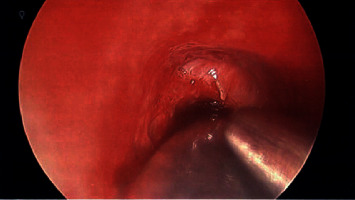
Tracheal stenosis inferior to the endotracheal tube balloon, without granuloma involvement.

**Figure 3:**
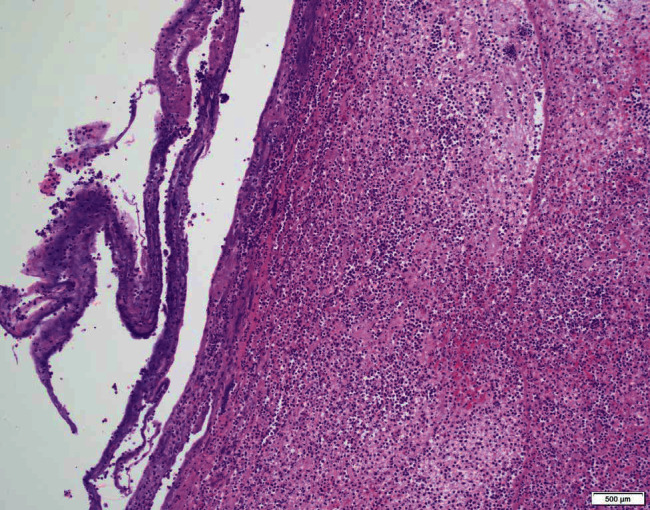
Specimen at 100x magnification demonstrating severe acute and chronic inflammatory infiltrate and granulation tissue. At the left of the image is a portion of fibrous tissue that encased the mass.

Our patient is a 40-year-old obese female with a complex medical history, including multiple cerebrovascular insults, goiter, and poorly controlled diabetes mellitus type II with a HbA1c of 9.8% and hypertension. To compound this, she had undergone multiple intubations, the longest of which lasted 5 days, and had previous tracheal stenosis requiring laser and balloon dilation only one month prior to presentation. She arrived at our institution for upper airway evaluation in the context of sudden onset respiratory distress requiring intubation at an outside hospital. Intraoperative laryngoscopy revealed a rapidly forming sessile transglottic tissue mass which not only filled the subglottic space superior to the cuff of the endotracheal tube but also began to “climb” up the sides of the tube itself (Figure 1). The tissue appeared adherent to the tube but was easily peeled away with forceps. Bronchoscopy beyond the endotracheal tube cuff demonstrated a patent airway without involvement of the granuloma (Figure 2). Cup forceps were used to remove the transglottic mass, and carbon dioxide (CO_2_) laser was used to further treat the tracheal stenosis. Balloon dilation was performed to 15 mm to re-establish patency, after which exposed cartilage was visible in the subglottic space. A tracheostomy was then performed given the unpredictable nature of the patient's airway. Final pathology demonstrated reactive tissue consistent with granuloma along with fibrinopurulent exudate and severe edema, measuring 5.0 × 2.2 × 0.8 cm in total (Figure 3). The patient had been lost to follow-up amidst the pandemic of coronavirus disease 2019 (COVID-19) and remains with tracheostomy tube with further treatment as anticipated.

## 3. Discussion

Our case represents an extraordinarily rapid growing and massive laryngeal granuloma. Of those who undergo endotracheal intubation, the majority will experience laryngeal erythema, mucosal ulceration, or granulation tissue [9]. However, for most patients, this will resolve within six weeks [6]. In one large case series of ninety-seven patients undergoing intubation longer than three days, forty-four developed a laryngeal granuloma and did so on in average within four weeks of extubation [6]. Despite an average of one month, development of granulomas in this cohort varied anywhere from two to ten weeks. Many smaller granulomas resolve on their own, often between eight to fourteen weeks postextubation, but larger granulomas often require surgical excision. The exuberant growth of our patient's granuloma may in part be due to female sex, previous prolonged intubations, and the exposed perichondrium visible in her trachea, but this is not a complete explanation of her unique reaction. While her granuloma was completely removed intraoperatively, her recurrence pattern has yet to be seen. Treatment of tracheal stenosis encompasses a wide and multidisciplinary array of options. These range from minimally invasive balloon dilation, to laser or cold knife widening, to tracheal resection and anastomosis. We chose to pursue carbon dioxide (CO_2_) laser ablation with balloon dilation due to previous response to these modes and overall poor health which made more invasive techniques a higher risk. The use of anti-inflammatory drugs, steroids, antibiotics, zinc sulfate, acid suppressive therapy, and surgery has been studied for preventing and/or treating laryngeal granuloma formation postextubation, but these methods have varying efficacy and no first-line treatment has been established [10]. A convergence of medical comorbidities including poor wound healing, chronic proinflammatory state, and iatrogenic manipulation of delicate mucosal sites may yield such a phenomenon. The considerable size of the granuloma tissue excised in this case and associated sudden onset of her symptoms make this case remarkable.

## 4. Conclusion

Our case highlights a rare laryngeal finding of a large granulation tissue mass causing sudden onset airway obstruction. A review of the literature identified that our patient had several documented risk factors for postintubation tracheal stenosis, but the large size of the granuloma and the short course in which it formed were complications that could not be foreseen. Most granulomas resolve on their own, but large ones often are indications for surgical intervention, as in this instance. We believe recognizing risk factors for postintubation tracheal stenosis early throughout a patients' treatment course may encourage more judicious observation helping to avoid unexpected airway compromise.
